# Research Progress on the Mechanism of Action and Screening Methods of Probiotics for Lowering Blood Lipid Levels

**DOI:** 10.3390/foods14091583

**Published:** 2025-04-30

**Authors:** Jingli Wang, Jieyu Chen, Mingkun Gao, Zijun Ouyang, Yanhui Li, Dong Liu, Mingjun Zhu, Haiyan Sun

**Affiliations:** 1 School of Food and Drug, Shenzhen Polytechnic University, Shenzhen 518055, China; wjl1582218@163.com (J.W.); chenjieyu@szpu.edu.cn (J.C.); kun502513@163.com (M.G.); ouyangzijun@szpu.edu.cn (Z.O.); liyanhui1@szpu.edu.cn (Y.L.); liudongsz@szpu.edu.cn (D.L.); 2 School of Biology and Biological Engineering, South China University of Technology, Guangzhou 510006, China

**Keywords:** hyperlipidemia, probiotics, lipid metabolism, screening, short-chain fatty acids

## Abstract

Hyperlipidemia is one of the most prevalent metabolic disorders worldwide. It is a significant risk factor for a range of cardiovascular diseases, including acute pancreatitis, fatty liver disease, atherosclerosis, and coronary heart disease. In clinical practice, the management of hyperlipidemia is hindered by numerous challenges. One of the critical issues is that traditional lipid-lowering drugs often require long-term or even lifelong administration, potentially inducing a range of adverse effects that compromise patient compliance and therapeutic efficacy. Therefore, there is an urgent need to develop safer and more effective strategies for the prevention and adjunctive treatment of hyperlipidemia with the aim of reducing the risk of disease and over-reliance on medication. Recent studies have revealed a close relationship between hyperlipidemia and related metabolic disorders involving gut microbiota dysbiosis, and the administration of probiotics has been shown to improve lipid metabolism homeostasis. This review summarizes the molecular mechanisms of probiotics in hyperlipidemia treatment and the latest advances in probiotic research on lipid metabolism, enumerates the experimental and clinical applications of probiotic-based therapies, introduces methods for screening and identifying probiotics with lipid-lowering functions, and, for the first time, summarizes the roles of emerging technologies such as functional genomics and in vivo zebrafish-on-a-chip models in studying the lipid-lowering efficacy of probiotics, providing insights for researchers. By facilitating a deeper understanding of the mechanisms whereby probiotics reduce blood lipid levels and furthering the development of multifaceted screening methods, we hope that we can achieve high-throughput and efficient screening of probiotics with lipid-lowering functions, thereby promoting the sustainable, high-quality, and rapid development of the probiotics industry.

## 1. Introduction

Hyperlipidemia, also known as high blood lipid levels, is a type of dyslipidemia characterized by an increase in the concentrations of total cholesterol (TC), low-density lipoprotein cholesterol (LDL-C), or triglycerides (TGs) in the plasma; a decrease in the concentration of high-density lipoprotein cholesterol (HDL-C); or a combination of these features [1]. Hyperlipidemia is a metabolic disorder and one of the most common chronic diseases. Excessive energy intake or lipid metabolism disorders can lead to hyperlipidemia. Hyperlipidemia is a significant risk factor for cardiovascular diseases such as atherosclerosis and coronary heart disease, as well as acute pancreatitis and fatty liver disease [2]. According to the Chinese Guidelines for Lipid Management (2023) [3], the diagnostic criteria for normal blood lipid levels in adults are as follows: TC < 5.2 mmol/L (200 mg/dL), TG < 1.7 mmol/L (150 mg/dL), LDL-C < 3.4 mmol/L (130 mg/dL), and HDL-C ≥ 1.0 mmol/L (40 mg/dL). When an individual’s lipid levels exceed these reference ranges for normal lipid profiles, their risk of developing hyperlipidemia increases significantly. According to the latest statistics from the Global Burden of Disease (GBD), hyperlipidemia caused 4.3 million deaths in 2019. The World Health Organization indicates that hyperlipidemia is one of the top five causes of death globally. Additionally, a 2021 study conducted on one million people in China revealed that approximately 33.8% of individuals aged 35–75 have dyslipidemia [4], and the average serum levels of TC, TG, and LDL-C in Chinese adults have been consistently rising. Moreover, because of the poor lifestyle habits and high-fat, high-sugar diets of many adolescents and children, the prevalence of dyslipidemia is increasing annually. Hyperlipidemia, traditionally considered a disease affecting the elderly, is becoming increasingly common in younger populations. Although many people are aware of this issue, the methods they adopt, such as taking weight-loss drugs or excessive dieting, can severely harm their health. Therefore, effective prevention and treatment of hyperlipidemia are becoming increasingly urgent.

It is well known that patients with hyperlipidemia require effective pharmacological treatment. Many lipid-lowering drugs are available on the market. However, these drugs need to be taken over the long term and render people prone to rebound effects after discontinuation, leading to significant side effects. Statins, one of the primary drugs used for hyperlipidemia treatment, specifically inhibit 3-hydroxy-3-methylglutaryl-CoA reductase (HMGCR), the rate-limiting enzyme in cholesterol synthesis. Nevertheless, 10% to 25% of patients experience statin-associated muscle symptoms (SAMSs), such as myalgia, myositis, and even rhabdomyolysis, during clinical treatment. Additionally, statins occasionally lead to hepatic dysfunction (elevated transaminase levels) [5]. Recent clinical studies have also indicated that long-term statin use may disrupt glucose metabolism, increasing the risk of new-onset diabetes. Unlike statins, fibrates and niacin primarily act by reducing serum TG levels. However, both classes of drugs frequently induce gastrointestinal adverse effects (e.g., diarrhea, nausea, vomiting, etc.), while niacin drugs may lead to skin flushing. Ezetimibe, which lowers LDL-C levels by inhibiting exogenous cholesterol absorption, may provoke muscle-related adverse reactions and allergic responses (e.g., rash, pruritus, etc.) [6,7]. Proprotein convertase subtilisin/kexin type 9 (PCSK9) inhibitors, a newer class of lipid-lowering agents, enhance hepatic LDL-C uptake, effectively reducing blood lipid levels. However, their high cost and requirement for subcutaneous injection limit their widespread use. In addition to post-disease treatment, effective prevention of hyperlipidemia is also a major challenge. The traditional methods of preventing hyperlipidemia mainly involve developing good lifestyle habits and a reasonable diet, which largely depend on individual self-discipline and are difficult to maintain and popularize. Thus, there is an increasing need for scientific nutritional strategies for preventing and adjunctively treating hyperlipidemia, thereby reducing the risk of this disease and over-reliance on medications.

While the aforementioned lipid-lowering drugs are effective, their adverse effects constrain their clinical utility, driving the exploration of alternative therapies and nutritional interventions. Related research can be traced back to the 1970s, when Mann et al. first reported that long-term consumption of lactobacillus-fermented dairy products was associated with lower serum cholesterol levels, providing a novel perspective for hyperlipidemia prevention and management [8,9]. Subsequently, in 2006, Ley et al. [10] discovered significantly higher Firmicutes/Bacteroidetes ratios in the gut microbiota of obese individuals, thereby establishing a link between gut dysbiosis and metabolic disorders. High-fat diets induce gut microbiota dysbiosis, which, in turn, has an impact on the progression of lipid metabolism by modifying the composition of bile acids and short-chain fatty acids (SCFAs).

Evidence from numerous clinical and experimental studies indicates that probiotics play an important role in regulating lipid metabolism. Through interactions with gut microbiota, probiotics and their metabolites contribute to regulating the lipid intake and metabolism of gut flora. Through clinical research, Khongrum et al. [11] found that intervention with *Lactobacillus paracasei* TISTR 2593 significantly reduced serum LDL-C levels and increased plasma apolipoprotein E levels in hypercholesterolemic patients, thereby reducing the risk of atherosclerosis. Furthermore, a clinical trial involving hyperlipidemic patients found that taking a mixed probiotic preparation not only reduced TC and LDL-C levels and increased HDL-C levels but also increased the abundance of beneficial bacteria, such as *Bifidobacterium animalis* and *Lactobacillus plantarum*, in the gut microbiota, further confirming the potential of probiotics in the field of lipid lowering and providing a new direction for the prevention of hyperlipidemia [12]. Many researchers, both domestically and internationally, are dedicated to uncovering the mechanisms through which probiotics lower blood lipid levels, but we still lack a systematic understanding of these mechanisms. Additionally, consumers seem to have reached a consensus regarding the health benefits of probiotics. According to the International Probiotics Association, China is the world’s second-largest probiotic consumer market, and the market size is expanding at a rate of 11–12%. The Prospective Industry Research Institute predicts that China’s probiotic market size will exceed CNY 190 billion by 2028. Faced with such a vast market demand, there is an urgent need to establish efficient and targeted models and methods for evaluating probiotics’ efficacy.

This review elucidates the multifaceted mechanisms through which probiotics modulate lipid metabolism. Furthermore, it provides a comprehensive review of the methods and models utilized both domestically and internationally for screening probiotics with lipid-lowering properties, discussing the advantages and limitations of various approaches. Additionally, this study highlights the most recent and promising strategies for the screening and efficacy evaluation of lipid-reducing probiotics. The aim of this work is to establish a theoretical foundation for the development of efficient and targeted methods for assessing the efficacy of probiotics.

## 2. Mechanisms of Probiotics in Regulating Lipid Metabolism

### 2.1. Assimilation and Adsorption of Lipids by Microbial Cells

The mechanisms through which probiotics regulate lipid metabolism are complex and multifaceted, with one of the most direct modes of action being the assimilation and adsorption of lipids. Probiotics can absorb cholesterol into their cells, which is subsequently excreted from the body via feces, thereby reducing the total circulating cholesterol levels. This process is known as the assimilation of cholesterol by probiotics [13]. In addition to assimilation, the cell membranes of probiotic bacteria also exhibit the ability to adsorb cholesterol. For instance, Liong et al. [14] observed cholesterol adsorbed onto the surfaces of bacterial cells using scanning electron microscopy. Similarly, Noh et al. [15] demonstrated that *Lactobacillus acidophilus*, when cultured in a cholesterol-supplemented medium, exhibited changes in cell membrane cholesterol content and increased resistance to ultrasonic lysis. This information suggests that cholesterol is either adsorbed onto or embedded within the cell membranes of lactic acid bacteria, altering the composition of fatty acids. Both the assimilation and adsorption of lipids by probiotic cells contribute to increasing the excretion of lipids from the intestinal tract, thereby playing a role in lipid metabolism regulation.

### 2.2. Regulation of Lipid-Metabolism-Related Transcriptional Factors

#### 2.2.1. Short-Chain Fatty Acids and Their Related Signaling Pathways

SCFAs, including acetate, propionate, and butyrate, are the end products of the fermentation of dietary fiber and resistant starch by gut microbiota. The health benefits of SCFAs are well established, as they have been shown to alleviate various diseases, including colorectal cancer, diabetes, and cardiovascular diseases [16]. Numerous studies have demonstrated that SCFAs, as key signaling molecules, play a regulatory role in energy metabolism, including lipid metabolism [17].

SCFAs contribute to appetite suppression by binding to specific SCFA receptors, GPR43 and GPR41, which promote the secretion of gut hormones such as leptin, glucagon-like peptide-1 (GLP-1), and peptide YY (PYY). These hormones enhance satiety, suppress appetite, and increase energy expenditure [18,19,20]. Beyond appetite regulation, SCFAs also inhibit lipid synthesis and promote fatty acid oxidation. For instance, Huo et al. [21] found that acetate supplementation significantly enhanced fatty acid *β*-oxidation in 3T3-L1 adipocytes, thereby reducing lipid accumulation. Similarly, Lin et al. [22] demonstrated that a mixture of acetate, propionate, and butyrate significantly lowered plasma cholesterol levels in rats. Interestingly, probiotics have been shown to modulate SCFA levels in the gut. Studies have revealed that the intake of probiotics increases the abundance of SCFA-producing bacteria and restores SCFA levels, which are significantly reduced by high-fat diets, to normal levels [23].

The AMP-activated protein kinase (AMPK) signaling pathway plays a prominent role in the SCFA-mediated regulation of lipid metabolism. SCFAs activate the G-protein-coupled receptors GPR41 and GPR43, inducing intracellular Ca^2+^ release [24] and modulating AMPK activity through the Ca^2+^/CAMKK*β* pathway [25]. The first important aspect in this regard is that peroxisome proliferator-activated receptor alpha (PPAR*α*), a downstream target of AMPK, has been found to promote fatty acid oxidation, a capacity that has been comprehensively documented. Huo et al. [21] demonstrated that intervention with *Bifidobacterium animalis* subsp. *lactis* A6, isolated from the feces of a centenarian, upregulated the expression of fatty acid *β*-oxidation-related proteins, including carnitine palmitoyltransferase 1 (CPT1), carnitine palmitoyltransferase 2 (CPT2), long-chain acyl-CoA dehydrogenase (LCAD), and medium-chain acyl-CoA dehydrogenase (MCAD), in the epididymal adipose tissue of mice. This effect was attributed to acetate produced by *B. animalis* subsp. *lactis* A6, which activated fatty acid *β*-oxidation via the GPR43-PPAR*α* signaling pathway. Second, AMPK signaling also regulates a series of lipid-synthesis-related genes. For example, *Lactobacillus plantarum* LP104 has been reported to activate AMPK signaling, significantly inhibiting the expression of phosphorylated acetyl-CoA carboxylase (ACC) and downregulating the expression of lipogenic proteins such as sterol regulatory element-binding protein-1 (SREBP-1) and fatty acid synthase (FAS) [26], thereby suppressing lipid synthesis at its source. Additionally, SCFAs mediate lipid degradation through the AMPK signaling pathway by modulating key rate-limiting enzymes in lipolysis, such as hormone-sensitive lipase (HSL) and adipose triglyceride lipase (ATGL).

In summary, SCFAs exhibit significant potential in terms of their lipid-lowering effects. However, because of the complexity of gut microbiota and signaling pathways, evaluating the lipid-lowering efficacy of lactic acid bacteria through SCFAs remains challenging. Future research is expected to address these challenges and provide further insights into the mechanisms underlying SCFA-mediated lipid metabolism regulation.

#### 2.2.2. Ferulic Acid Esterase

Ferulic acid, a phenolic compound predominantly found in cereals and vegetables, has been found in numerous animal models and clinical trials to significantly reduce serum levels of TC, TG, and LDL-C while markedly increasing HDL-C levels [27,28]. Further studies have revealed that ferulic acid acts as a competitive inhibitor of HMGCR, the rate-limiting enzyme in de novo cholesterol synthesis, thereby suppressing cholesterol production at its source [28]. Additionally, ferulic acid is an activator of PPAR*α*, promoting fatty acid oxidation [29].

However, ferulic acid is primarily embedded within the plant cell walls of cereals and vegetables, making it difficult to digest and utilize. Ferulic acid esterase (FAE), an enzyme capable of hydrolyzing ester bonds in ferulic acid esters, disrupts the internal reticular structure of plant cell walls, thereby releasing ferulic acid. Certain lactic acid bacteria, such as *Lactobacillus plantarum*, *Lactobacillus fermentum*, *Lactobacillus acidophilus*, and *Lactobacillus johnsonii*, have been shown to produce FAE [30]. Nevertheless, the efficacy of this enzyme is closely related to dietary habits. If one’s daily diet lacks foods rich in ferulic acid, the effects of feruloyl esterase may be limited. Therefore, FAE-producing lactic acid bacteria should be combined with prebiotics rich in ferulic acid to achieve optimal results.

#### 2.2.3. Conjugated Linoleic Acid

Lactic acid bacteria have the ability to convert polyunsaturated fatty acids (primarily linoleic acid and linolenic acid) ingested from the diet into conjugated fatty acids, specifically through the isomerization of linoleic acid and linolenic acid into conjugated linoleic acid (CLA) and conjugated linolenic acid (CLNA). Extensive research has demonstrated that dietary CLA can modulate the composition of fatty acids in human plasma, reducing the levels of TC, TG, LDL-C, and VLDL-C [31,32]. Moreover, the physiological functions of CLA produced by lactic acid bacteria are primarily attributed to cis-9,trans-11-CLA (c9,t11-CLA) and trans-10,cis-12-CLA (t10,c12-CLA) [33]. Among these, t10,c12-CLA is the second most abundant after c9,t11-CLA and plays a significant role in regulating lipid metabolism. t10,c12-CLA can promote the secretion of GLP-1 through G protein-coupled receptors 40/120 (GPR40/120) and enhance lipid breakdown [34]. Additionally, t10,c12-CLA can modulate the activity of PPAR*α* and sterol regulatory element-binding proteins (SREBPs), thereby enhancing fatty acid oxidation and lipid synthesis [35,36].

From this perspective, both CLA and SCFAs are fatty acids, and their lipid-lowering mechanisms are quite similar. However, the conditions required for the production of CLA are relatively stringent, and this process’s mechanisms of action are not yet fully understood. Therefore, compared to SCFAs, the CLA level remains a challenging criterion for the efficient screening of lipid-lowering probiotics.

### 2.3. Influence on Lipid Transport and Absorption

#### 2.3.1. Bile Salt Hydrolase

Bile salt hydrolase (BSH) is an intracellular enzyme produced during the growth and metabolism of gut microbiota. Studies have found that certain probiotics can produce BSH, which helps reduce serum cholesterol. The lipid-lowering mechanism of BSH is closely related to the activity of bile acids in the body [37,38]. Bile acids are synthesized in the liver and stored in the gallbladder. When a human ingests food, their gallbladder secretes bile to aid digestion and absorption. Since dietary lipids are insoluble in water, and they require emulsification by bile to form stable micelle solutions, which can then be better absorbed by intestinal epithelial cells. However, the BSH expressed by probiotics catalyzes the hydrolysis of conjugated bile salts into free bile salts and amino acid residues. Free bile salts are less soluble than conjugated bile salts, making them less likely to be reabsorbed and, thus, easier to excrete from the body. On the one hand, as bile salts are a crucial component of bile, a reduction in their levels inevitably leads to decreased bile levels in the intestine, thereby affecting lipid absorption [37,39]. On the other hand, the excretion of bile salts reduces the reabsorption of bile acids in the intestine. The bile acid pool in the body is fixed, thus prompting the liver to utilize cholesterol to resynthesize bile acids via cholesterol 7*α*-hydroxylase (CYP7A1), indirectly lowering plasma cholesterol levels. Therefore, bile salt hydrolase activity is also considered an important criterion for selecting lipid-lowering probiotics. Numerous studies have demonstrated that strains exhibiting higher BSH activity, such as *Lactobacillus plantarum* Y15 [40] and *Lactobacillus plantarum* ECGC 13110402, possess superior potential for lipid-lowering action [41]. Notably, the hypolipidemic efficacy of *L. plantarum* ECGC 13110402 has been clinically validated. However, strains with BSH activity are typically found among intestinal-derived strains, making it difficult to isolate BSH-expressing probiotics in environments lacking bile salts.

#### 2.3.2. Exopolysaccharides

Exopolysaccharides (EPSs) are extracellular macromolecules secreted by microorganisms in the form of tightly bound capsules or loosely attached slime layers. Based on their distribution, EPSs can be classified into capsular polysaccharides and slime polysaccharides. Capsular polysaccharides adhere to the peptidoglycan layer of the microbial cell wall, while slime polysaccharides are secreted into the growth environment. Probiotic EPS is a prominent research focus and is widely utilized to modify the rheological properties and texture of fermented dairy products. In addition, Nakajima et al. [42] discovered that EPS produced by lactic acid bacteria can regulate the cholesterol balance. Over a span of years, Maeda and London et al. [43] confirmed this phenomenon through mouse experiments. It has been hypothesized that, because of the structural similarity between EPS and dietary fiber, EPS may regulate cholesterol levels through analogous mechanisms. These mechanisms include inhibiting the absorption of cholesterol and fats, promoting the metabolism of cholesterol into bile acids, and enhancing intestinal motility to increase cholesterol excretion. Some lactic acid bacteria EPSs, such as *β*-glucans, are generally not digested or absorbed by the human body and modulate the cholesterol balance through mechanisms akin to those of dietary fiber. Additionally, certain capsular EPSs from lactic acid bacteria can bind bile acids and facilitate their excretion, thereby promoting the synthesis of bile acids from cholesterol.

#### 2.3.3. Regulation of the Expression of Relevant Transport Proteins

**Figure 1:**
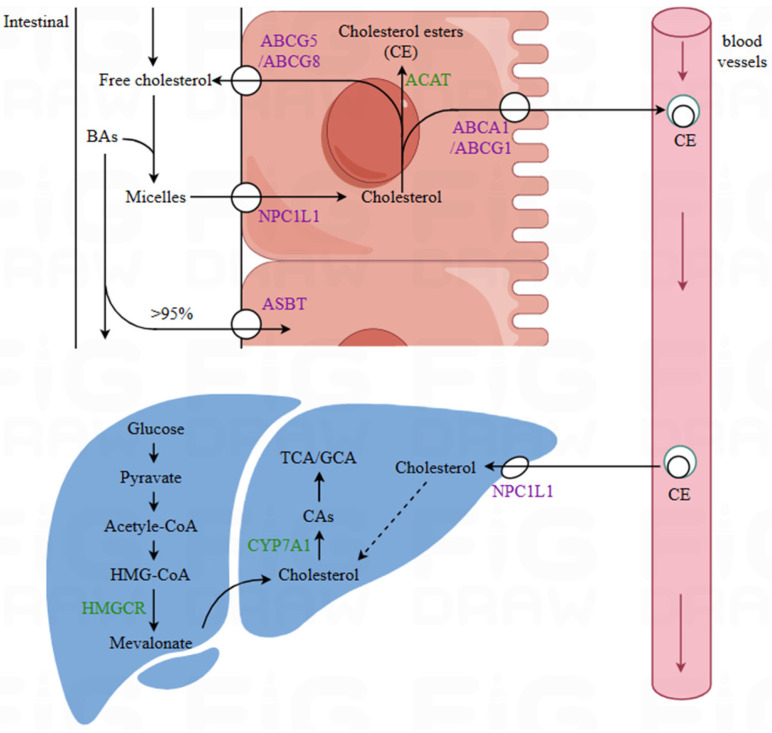
Cholesterol absorption and synthesis. BAs—bile acids; NPC1L1—Niemann–Pick C1-like 1; ABCG5/ABCG8—ATP-binding cassette G5/G8; ABCA1/ABCG1—ATP-binding cassette A1/G1; ASBT—apical sodium-dependent bile acid transporter; CE—chylomicron; HMGCR—3-hydroxy-3-methylglutaryl-CoA reductase; CYP7A1—cholesterol 7*α*-hydroxylase; CAs—cholic acids; TCA/GCA—taurocholic acid/glycocholic acid; ACAT—Acyl-CoA cholesterol acyltransferase. All the black solid arrows in the figure represent the metabolic effects and transport pathways of cholesterol, the dashed arrows indicate the incorporation of exogenous cholesterol into liver cholesterol, and the red realization arrows indicate the direction of blood flow in the blood vessels.

Cholesterol absorption and transport involve numerous transport proteins. As illustrated in Figure 1, the uptake of cholesterol in the intestine primarily depends on Niemann–Pick C1-like 1 (NPC1L1). A portion of the cholesterol taken up by intestinal epithelial cells is transported back to the intestinal lumen via ATP-binding cassette members G5 and G8 (ABCG5/G8). The remaining cholesterol is esterified into chylomicrons by acyl-CoA cholesterol acyltransferase (ACAT). The cholesterol in chylomicrons is transported to the liver via the bloodstream. Newly synthesized cholesterol in the liver and cholesterol from chylomicrons are transferred to peripheral cells in the form of LDL-C through the low-density lipoprotein receptor (LDL-R). Subsequently, ATP-binding cassette A1/G1 (ABCA1/G1) mediates the efflux of excess cholesterol from cells to apolipoprotein A-I (ApoA-I), thereby forming HDL-C. HDL-C is subsequently transported back to the liver via scavenger receptor class B type I (SR-BI) and excreted [44,45].

Lactic acid bacteria may influence cholesterol metabolism and absorption processes by regulating the gene expression of cholesterol transport proteins. ABCG5/G8 can transport a portion of the cholesterol taken up by intestinal epithelial cells back to the intestinal lumen, thereby limiting cholesterol absorption. ABCA1/G1 can increase cholesterol efflux. NPC1L1 is abundantly expressed in both the small intestine and the liver. In the small intestine, it mediates the absorption of cholesterol from the intestinal lumen, while in the liver, it facilitates the uptake of circulating cholesterol from the bloodstream [46]. In a study by Zhao et al. [47] on the mechanism by which *Lactobacillus plantarum* WLPL21 alleviates hypercholesterolemia, it was found that *Lactobacillus plantarum* WLPL21 significantly upregulates the expression of ABCA1 and ABCG5/G8 in the ileum and liver and significantly downregulates the expression of NPC1L1.

The hypolipidemic effects of probiotics result from the combined actions of multiple pathways, with potential synergistic interactions occurring among different mechanisms. Based on our current understanding of probiotic-mediated lipid-lowering mechanisms, the prerequisite for probiotics to exert metabolic effects through their bioactive metabolites is successful intestinal colonization. Probiotic-derived SCFAs facilitate colonization by reducing local intestinal pH, thereby inhibiting the growth of pathogenic bacteria and securing ecological niches [48]. Additionally, the bile-salt-rich intestinal environment serves as a selective pressure for microbial survival, wherein BSH-active probiotics gain competitive advantages [36]. These colonization-enabling mechanisms may synergize with other lipid-modulating pathways to enhance probiotics’ persistence and hypolipidemic efficacy. However, research on such mechanistic synergies remains scarce. Current studies predominantly focus on single strains or isolated mechanisms, and even when multiple pathways are implicated in particular strains, their interrelationships remain poorly characterized. This knowledge gap not only represents a crucial direction for future investigation but may also inform the development of integrated screening approaches for identifying high-efficacy lipid-lowering probiotics through mechanistic synergy-based selection models.

## 3. Research on Probiotic-Mediated Lipid-Lowering Effects

Based on the current state of research into the mechanisms through which probiotics reduce blood lipid levels, the specific lipid-lowering effects of probiotics are garnering an increasing amount of research attention. Currently, scientists worldwide are focusing on isolating probiotic strains from traditional fermented foods (such as kimchi, fermented dairy products, and red yeast rice wine) and the gut microbiotas of specific populations and subsequently conducting efficacy studies. While most probiotics remain in the preclinical research stage, certain strains have progressed to clinical trials and demonstrated significant improvements in lipid profiles (Table 1). A connection is now being established between the fundamental research on the lipid-lowering mechanisms of probiotics and their clinical therapeutic applications.

Both animal studies and clinical trials have demonstrated the considerable potential of probiotics in the treatment of hyperlipidemia. In animal experiments, certain probiotic strains exhibited lipid-lowering effects comparable to those of conventional lipid-lowering drugs (e.g., atorvastatin and simvastatin). However, due to the lack of positive controls in clinical studies, direct efficacy comparisons remain challenging. Notably, one clinical trial investigating the combined use of probiotics and atorvastatin revealed that probiotic supplementation enhanced gut microbiota diversity [59]. This finding suggests that probiotics may serve as an ideal adjuvant therapy for statin-intolerant patients or those with mild hyperlipidemia. Nevertheless, a critical scientific issue must be addressed before broader clinical application: the strain-specific nature of probiotics leads to significant variations in efficacy, yet there are currently no systematic and efficient standardized screening methods available. Therefore, a comprehensive understanding of the existing screening approaches and emerging screening models is necessary.

## 4. Screening Methods for Lipid-Lowering Probiotics

### 4.1. Traditional Screening Techniques for Lipid-Lowering Probiotics

#### 4.1.1. In Vitro Cholesterol and Triglyceride Reduction

The most traditional method for screening probiotics with cholesterol- and triglyceride-lowering capabilities involves supplementing liquid culture media with additional cholesterol/triglycerides and measuring the reduction in cholesterol/triglyceride levels in the medium to determine the rate of degradation by the strains. In vitro cholesterol/triglyceride reduction experiments directly reflect the absorption of cholesterol and triglycerides by probiotics as well as the co-precipitation of cholesterol. This method has been the most commonly used approach for screening lipid-lowering probiotics in the past [60,61,62,63], and many still employ this method to date. To some extent, this method can also reduce the likelihood of missing potential strains.

However, the mechanisms of cholesterol removal vary among different strains [61]. For example, studies have found that *Lactobacillus plantarum* Lp501 and Lp529 remove similar amounts of cholesterol, but the proportions of absorbed and precipitated cholesterol differ significantly [64]. Additionally, research has shown that most of the cholesterol absorbed into the cells is not degraded but instead fluctuates with changes in the concentration gradient of cholesterol inside and outside the cells. Therefore, cholesterol absorbed by probiotics may be re-excreted. Furthermore, while probiotics hydrolyze triglycerides into glycerol and free fatty acids in the intestine, these products can be reabsorbed by intestinal epithelial cells and re-synthesized into triglycerides. Thus, probiotics screened for their ability to hydrolyze triglycerides may not effectively reduce triglyceride intake. In addition to these issues, using this method to screen for lipid-lowering probiotics requires measuring the cholesterol/triglyceride degradation rate for each individual strain. This results in a substantial workload and makes high-throughput screening impractical.

#### 4.1.2. Qualitative and Quantitative Assays for BSH Activity

In simple terms, the qualitative assay for BSH activity is based on the principle that free bile salts are less soluble than conjugated bile salts. When strains are cultured on MRS agar medium supplemented with tauro/glyco-(deoxy)cholate, intracellular BSH hydrolyzes tauro/glyco-(deoxy)cholate into free bile acids. These free bile acids precipitate because of their low solubility [65]. As a result, strains with BSH activity form precipitation circles around their colonies, and the diameters of these circles can roughly reflect the level of BSH activity. Table 2 summarizes specific methods for qualitative BSH assays.

The qualitative BSH assay can be used to screen many strains for BSH activity but cannot quantify the enzyme’s activity. Currently, the quantitative methods for BSH activity determination primarily include the ninhydrin colorimetric method and high-performance liquid chromatography (HPLC) [66,67]. Both methods are based on the hydrolysis of conjugated bile acids into free bile acids and amino acids. The ninhydrin colorimetric method reflects BSH activity by measuring the number of amino acids produced, while HPLC measures the reduction in conjugated bile acids to reflect BSH activity. In the ninhydrin colorimetric method, bacterial cells are ultrasonically lysed to extract the BSH enzyme solution, which is then reacted with conjugated bile acids. After the reaction, ninhydrin reagent is added for color development, and the amino acid content is determined by measuring absorbance [66]. In contrast, HPLC is simpler to use and provides more accurate measurements. However, both quantitative methods share a common limitation: The in vitro growth environment differs from in vivo conditions, rendering the enzyme activity measured in vitro less relevant for predicting in vivo function. Therefore, in many cases, instead of quantifying bile salt hydrolase (BSH) activity, it is sufficient to confirm that a strain possesses it.

Compared to in vitro methods for detecting cholesterol and triglyceride reduction, the screening approach based on BSH activity is simpler and more targeted, making it highly suitable for large-scale preliminary screening. As indicated by the aforementioned BSH-mediated lipid-lowering mechanisms, BSH activity is widely recognized as one of the critical criteria for screening probiotics with potential lipid-lowering effects. However, the impact of BSH on the host remains controversial. While BSH can alleviate hyperlipidemia by reducing serum TC levels, it may also contribute to lipid malabsorption and steatorrhea and, in more severe cases, exacerbate gastrointestinal disorders such as Crohn’s disease and ulcerative colitis. Therefore, the long-term safety of probiotics exhibiting BSH activity is a significant consideration in the screening process for lipid-lowering probiotics.

#### 4.1.3. Cell Models

The human hepatocellular carcinoma cell line HepG2 offers advantages such as a short growth cycle, high differentiation stability, and infinite proliferation. It can express enzymes related to lipid metabolism, such as HMGCR and triglyceride lipase [68]. Therefore, HepG2 cells are widely used in research areas such as lipid lowering, anti-hepatoma, and anti-steatosis [67]. A lipid accumulation model in HepG2 cells can be established by inducing palmitic acid or linoleic acid to the cells, and the success of the model can be verified using lipid analysis kits and Oil Red O staining. If the accumulation of TG and the area of lipid droplets in the cells decrease after treatment with the fermentation supernatant or cell lysate of probiotics, it can be preliminarily concluded that the strain has lipid-lowering effects. Further investigation into the specific lipid-lowering mechanisms can be conducted by measuring changes in the expression of lipid-metabolism-related genes before and after intervention. For example, Tang et al. [69] used this method to discover that the cell-free extract of *Lactobacillus acidophilus* NX2-6 reduced TG and LDL-C levels in HepG2 cells and significantly influenced the expression of lipid-metabolism-related genes such as SREBP-1c, FAS, and CPT1.

The 3T3-L1 preadipocyte cell line is derived from Swiss mouse embryos. The advantages of the 3T3-L1 cell line include stable passage and efficient differentiation into adipocytes, thus more systematically simulating the process of lipid metabolism. Therefore, the 3T3-L1 cell line is a widely recognized model for studying lipid metabolism, lipid-lowering, and anti-obesity drugs [70,71]. When using this cell line for lipid metabolism research, appropriate inducers are similarly employed to stimulate 3T3-L1 cells, causing them to differentiate into adipocytes filled with lipid droplets. Changes in lipid droplet content in adipocytes before and after intervention can be visually observed using Oil Red O staining. For instance, Won et al. [71] isolated 15 strains from Korean kimchi and found that *Lactobacillus sakei* ADM14 significantly reduced TG levels in 3T3-L1 cells while inhibiting the expression of adipogenesis-related genes, such as PPAR*γ*, C/EBP*α*, aP2, and FAS.

#### 4.1.4. High-Fat-Diet Rat and Mouse Models

Rats exhibit strong resistance to stress, excellent model stability, high reproducibility, and sufficient blood volume for detecting multiple indicators. Therefore, rats are currently the most widely used animal models (both domestically and internationally) for studying human lipid metabolism. The 2023 “Functional Testing and Evaluation Methods for Health Foods” provides detailed standard testing methods for maintaining healthy blood lipid levels, specifically using rat models. Hsieh et al. [72] found that both live and heat-inactivated *Lactobacillus reuteri* GMNL-263 reduced weight gain in a high-fat-diet (HFD) mouse model and significantly decreased the increase in lipid distribution in the serum and liver. Further investigation revealed that the intake of this probiotic successfully reversed the HFD-induced upregulation of adipogenesis-related genes in the liver. Zhang et al. [73] carried out an intervention using Sprague–Dawley rats involving fermented products of *Lactobacillus plantarum* dy-1, improving lipid composition and regulating lipid metabolism by modulating the expression of lipid-metabolism-related microRNAs (miRNAs).

Compared to rat models, mice are cheaper and easier to raise, exhibit stable blood lipid levels, and are easier to handle experimentally, so they are commonly used in studies screening lipid-lowering traditional Chinese medicines and probiotics [49,74,75]. The most common method for inducing hyperlipidemia in mouse models, specifically C57BL/6J mice, is long-term feeding with a high-fat diet. Wang et al. [75] evaluated the lipid-lowering effects of five different species of lactic acid bacteria using high-fat-diet-induced-hyperlipidemia C57BL/6J mice. The results showed that all five strains significantly reduced serum TC, TG, and LDL-C levels and increased HDL-C levels. Additionally, they had markedly smaller adipose tissue and alleviated hepatocyte steatosis and lipid vacuolation.

#### 4.1.5. High-Fat-Diet Zebrafish Model

Approximately 70% of the zebrafish genome shares homology with the human genome [76]. Additionally, the structure and function of the zebrafish intestine are similar to those of mammals, and their behavioral patterns and endocrine responses often align with clinical data [77]. Furthermore, zebrafish lipid metabolism is comparable to that of mammals such as rats, mice, and humans [78]. Zebrafish also exhibit rapid reproduction and development, making them suitable for high-throughput testing [77]. Beyond these physiological advantages, zebrafish offer unique experimental benefits. These include a powerful genetic manipulation system, a wide range of available transgenic models, the capacity to facilitate automated and species-specific behavioral analysis for gut microbiome–brain axis evaluation, and transparency during early development, enabling live imaging. Zebrafish are increasingly being used as a simple model organism in embryology, molecular genetics, and in vivo high-throughput drug screening. For example, Chen et al. [79] investigated how *Bacillus licheniformis* FA6 regulates lipid metabolism in zebrafish. They found that FA6 promoted lipid accumulation in zebrafish, with further studies revealing that it enhanced lipid synthesis while inhibiting fatty acid *β*-oxidation. Silvia et al. [80] successfully modulated the gut microbiota of zebrafish using *Lactobacillus rhamnosus*, increasing the abundance of Firmicutes and thereby downregulating genes related to cholesterol and triglyceride synthesis, ultimately reducing TC and TG levels. The authors of this study successfully utilized a high-fat zebrafish model to reveal the potential lipid-lowering effects of probiotics.

However, the zebrafish model also has limitations in terms of probiotic functional screening. The composition of the zebrafish gut microbiota differs significantly from that of mammals, with Proteobacteria dominating (76–82%) [81], whereas Firmicutes are predominant in mammals. Given that one of the primary mechanisms of probiotics is the modulation of the gut microbiota, the relevance of probiotic-induced changes in the zebrafish gut microbiome to mammals remains to be further investigated. Additionally, while the small size of zebrafish facilitates high-throughput testing, it also limits the collection of organ samples and the quantity of data that can be obtained.

### 4.2. Advanced Screening and Prediction Technologies

In recent years, research on the mechanisms of probiotics with lipid-lowering functions has increased significantly. However, traditional screening techniques for lipid-lowering probiotics are still hindered by drawbacks such as complex procedures, high workloads, poor reproducibility, and difficulties in standardizing methods. To address these issues, many researchers are dedicating their efforts to developing more efficient screening methods for probiotics. Although the prediction and screening methods mentioned below have not yet been applied to the screening of lipid-lowering probiotics, they hold potential in terms of their principles and objectives. It is hoped that these methods will provide valuable insights for the future development of screening technologies for lipid-lowering probiotics.

#### 4.2.1. Digitalized Live Zebrafish-on-a-Chip System

It is well known that in vitro models save time and are high-throughput and highly effective, making them suitable for the initial stages of screening lipid-lowering probiotics, particularly for the rapid screening of large samples. However, individual in vitro models are insufficient for reflecting the complex lipid metabolism processes in the human body. Therefore, simple model organisms such as zebrafish, which share high homology with humans in terms of metabolism, genetics, and physiology, can be utilized to bridge the gap between in vitro models and traditional animal experiments. Numerous studies have shown that changes in environmental substances can affect microbial composition, and the gut microbiota significantly influences the nervous system and development of zebrafish [82,83,84]. Consequently, a novel screening method has been developed to predict the functions of drugs or probiotics by analyzing and statistically evaluating the central nervous system of zebrafish. This method has already been applied to the prediction of the efficacy of antiepileptic drugs. For instance, Lin et al. [85] employed microfluidic chip technology to achieve efficient whole-brain imaging of zebrafish and obtained high-throughput brain activity maps. These brain activity maps were functionally classified and ranked using machine learning, and the potential efficacy of various substances was predicted based on the similarity of their activity maps to those of clinically used drugs. In this study, the authors selected 179 clinically used drugs targeting the central nervous system, collected whole-brain activity map data from zebrafish treated with these drugs, and established a dataset. Based on this dataset, they designed a clustering algorithm to determine the intrinsic consistency among samples, thereby creating a predictive and screening platform for drug functions. To validate the effectiveness of the platform, the authors applied the same strategy to analyze 121 non-clinically used drugs and predicted their potential antiepileptic effects. They then selected the top 14 drugs from the classification process and validated their efficacy using a zebrafish epilepsy model, finding that 7 of these drugs exhibited antiepileptic activity. This predictive platform successfully linked the therapeutic effects of drugs directly to physiological changes in the central nervous system of the model organism, significantly improving screening efficiency. Furthermore, Tang et al. [86] combined microfluidic technology with hydrogel encapsulation methods to achieve rapid zebrafish immobilization, generating “fish capsules”. The immobilized zebrafish were placed on a microdroplet array chip, enabling not only rapid parallel drug administration but also microscopic imaging of internal structures such as the central nervous and cardiovascular systems. Using this approach, the authors successfully evaluated the effects of neuroactive drugs, cardiovascular drugs, and combinations of two drug groups.

Although there are no specific examples of using the digitalized live zebrafish-on-a-chip system for screening lipid-lowering probiotics, these studies demonstrate its immense potential in drug and functional probiotic screening. However, several questions remain to be addressed. While the brain activity and heartbeat maps of zebrafish show clear correlations with central nervous system drugs, their relevance to lipid-lowering drugs has not been studied. In the future, in addition to brain activity and heartbeat maps, behavioral maps of zebrafish could also be incorporated into research.

#### 4.2.2. Functional Genomics Analysis of Probiotics

In addition to utilizing simple model organisms to assist in the screening of lipid-lowering probiotics, another highly promising strategy is the mining of functional genes related to lipid lowering. With the maturation of whole-genome sequencing technology and the widespread use of computational analysis systems, functional genomics pertaining to probiotics has gradually demonstrated its advantages. The functional genomics analysis of probiotics is based on large-scale genomic data obtained through sequencing, and it leverages public databases (such as KEGG) to annotate potential functional genes, thereby generating functional gene maps. Subsequently, artificial intelligence is employed to analyze and learn from these gene maps to predict and screen other probiotics that may possess corresponding functions [87]. While the traditional screening methods mentioned in Section 2.1 still cannot escape the need for cumbersome manual operations, functional genomic analysis can significantly improve the efficiency of screening lipid-lowering probiotics.

Although a mature functional gene screening platform for probiotics has not yet been established, Zhang Heping’s research team has successfully developed a probiotic prediction and screening platform (iProbiotics). Their construction strategy is similar, combining genomic databases with computational algorithms. This research team integrated genomic data from 239 probiotic strains and 412 non-probiotic strains and identified a series of core functional genes associated with probiotic characteristics. Based on this foundation, they employed computational analysis to predict whether specific strains possess probiotic properties [88]. However, the capabilities of this probiotic prediction and screening platform remain limited, as it cannot yet directly link different core functional genes to specific effects such as lipid lowering or blood sugar reduction. In addition, there is a case for a more detailed and practical application. Sun et al. [89] selected four strains of *Lactobacillus plantarum* (C232, E932, D444, and B652) that exhibited the best performance in terms of acid tolerance, bile salt tolerance, survival ability in simulated gastrointestinal environments, and antioxidant capacity, respectively, from 44 strains. They then performed whole-genome sequencing on these strains. Using *Lactobacillus plantarum* ST-III, which has antimicrobial properties, strong colonization ability, and cholesterol-lowering effects, as a reference strain, the authors compared the similarities and differences in their genomes. The authors found that the presence of genes encoding cell surface proteins, antioxidant enzymes, antimicrobial peptides and bacteriocins, stress response proteins, and cholesterol-lowering factors (such as genes related to ABC transporters for sulfonated lipids) was closely associated with the distinct functional phenotypes of the strains.

From the above studies, it is evident that functional genomics research on probiotics is still in its early stages but has broad application prospects and value. However, many challenges remain to be addressed in this process, one of which is that gene expression and regulation can also influence the specific functions of probiotics. Nevertheless, this fact does not diminish the value of functional genomics in the prediction and screening of lipid-lowering probiotics.

## 5. Conclusions

In summary, the mechanisms through which probiotics exert lipid-lowering effects are diverse and complex. This fact has driven the development of various screening techniques and predictive methods. However, both traditional screening technologies for lipid-lowering probiotics and emerging functional screening and prediction approaches have their respective advantages and limitations. Therefore, the further exploration of screening strategies for lipid-lowering probiotics necessitates the integration of both methodologies. As previously discussed, molecular and cellular evaluation models can be developed based on the mechanisms of action of lipid-lowering probiotics. These models, when combined with simple model-organism-based evaluation systems and validated through traditional animal experiments, can establish a high-throughput preliminary screening method for probiotics. Future research urgently requires further experiments to explore and compare the relationships among different in vitro models, zebrafish models, rat and mouse models, and functional genomics. Additionally, beyond the aforementioned functional evaluation experiments, a comprehensive series of safety assessments and physiological–biochemical characteristic evaluations should be conducted during in vitro probiotic screening. These include assessments of acid tolerance, bile salt tolerance, adhesion capacity, antibiotic resistance, and acute toxicity to ensure that the probiotics can successfully colonize and function in the human gastrointestinal tract without posing health risks. The application of probiotics is still hindered by several limitations, which primarily manifest in the following three aspects. First, as previously mentioned, the strain-specific effects of probiotics remain a significant challenge. Establishing standardized screening methods will be the most effective approach to addressing this issue. Second, probiotics are highly susceptible to environmental factors during production and storage, necessitating the development of high-density fermentation techniques and high-viability preservation technologies to enhance production stability. Third, the probiotic industry currently lacks a unified regulatory framework, primarily because of the absence of standardized screening methods, insufficient clinical evidence, and other contributing factors. To overcome the bottlenecks in probiotic applications, it is essential to advance synergistically in three key areas: establishing standardized screening methods, developing high-stability production technologies, and improving industry regulatory frameworks. As screening technologies for lipid-lowering probiotics mature, the era of true high-throughput probiotic screening will creep ever closer. The aim of this review is to provide insights into high-throughput screening strategies for lipid-lowering probiotics and inspire the design of screening methods and lipid-lowering models for functional probiotics.

## 6. Methods

This review was conducted according to the PRISMA guidelines. We systematically searched multiple databases, including PubMed, Web of Science, and the China National Knowledge Infrastructure (CNKI). The search timeframe spanned from the inception of each database to 2024. The keywords used included “hyperlipidemia”, “probiotics”, “lactic acid bacteria”, “Lactobacilli”, “Bifidobacterium”, “blood lipids”, “cholesterol”, “triglycerides”, “lipid metabolism”, “mechanism”, “pathway”, “bile salt hydrolase”, “gut microbiota”, “clinical trial”, “mice”, “rats”, “zebrafish”, and their combinations. We included the following types of publications: clinical trials, randomized controlled trials, narrative reviews, systematic reviews, and meta-analyses. The initial search yielded 2356 articles. After screening the titles and abstracts, we excluded 2269 articles as they did not meet the eligibility criteria. Subsequently, we performed full-text screening of the remaining 89 articles and ultimately included 6 randomized controlled trials (RCTs) and 8 animal studies for the core analysis. Narrative reviews, systematic reviews, and meta-analyses meeting the eligibility criteria from the remaining literature were utilized to supplement mechanistic discussions and background contextualization (the exact number was not quantified).

**Figure 2:**
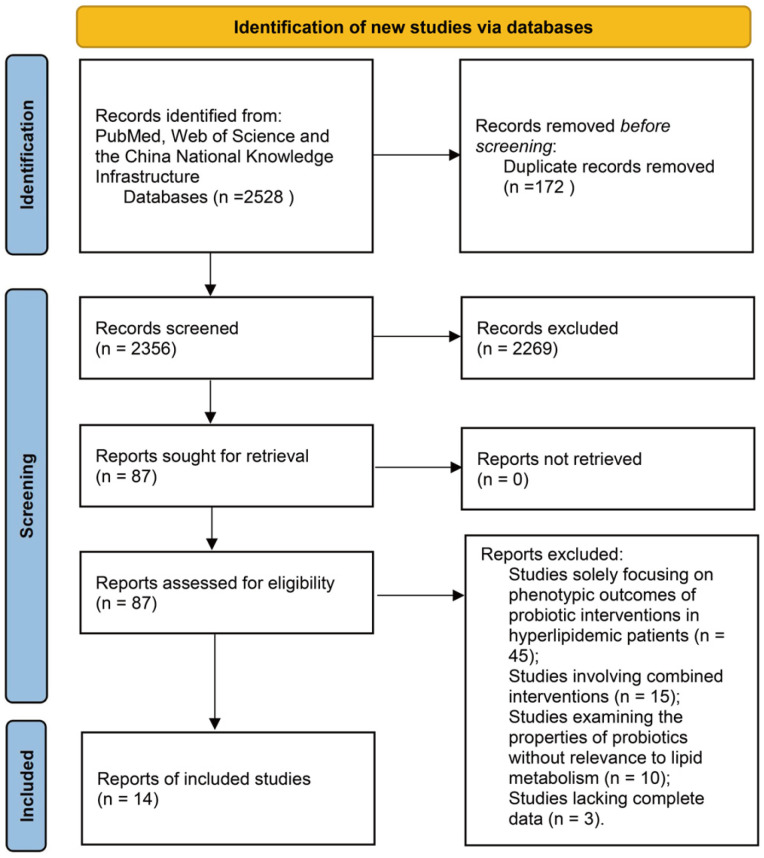
Search strategy.

The inclusion criteria were as follows: (1) studies investigating the effects of probiotics on blood lipid levels, the mechanisms of probiotics in lipid lowering, and the screening of lipid-lowering probiotics; (2) studies published in English; and (3) for the RCTs and animal studies included, the provision of at least one of the following complete datasets: (i) means ± standard deviations of blood lipid levels pre-intervention and post-intervention, (ii) differences in lipid changes between intervention and control groups, or (iii) the inclusion of a positive control group with statistical difference analysis. The exclusion criteria were as follows: (1) studies solely focusing on the phenotypic outcomes of probiotic interventions applied to hyperlipidemic patients; (2) studies involving combined interventions of probiotics and other active substances; and (3) studies examining the properties of probiotics without discussing their relevance to lipid metabolism. The flowchart of the database search process is presented in Figure 2.

We analyzed the included studies. Given the broad scope of probiotic research, the search strategy was designed to be flexible, which may have introduced certain limitations. To address this, we supplemented the search by tracking references from key articles to ensure the inclusion of additional relevant studies.

We conducted a risk-of-bias assessment for the included clinical trials and animal studies. For preclinical studies, the Cochrane Risk of Bias Tool (RoB 2.0) was used to evaluate RCTs, covering five domains: randomization process, deviations from intended interventions, missing outcome data, measurement of the outcome, and selective reporting. The assessment results are presented in Supplementary Table S1. For animal studies, the Systematic Review Centre for Laboratory Animal Experimentation (SYRCLE) risk-of-bias tool was applied, covering selection bias, performance bias, detection bias, attrition bias, reporting bias, and other biases. The evaluation results are shown in Supplementary Table S2.

## Figures and Tables

**Table 1:** Effect of probiotic strains on hyperlipidemia.

Study Type	Probiotic Strain	Subjects	Intervention Duration	Key Outcomes	Reference
Animal	*Lactobacillus plantarum* HAC01	High-fat-diet (HFD)-fed C57BL/6J mice	8 weeks	↓ Serum TC and TG levels (TC: ↓ 19.75%; TG: ↓ 16.67% vs. model)	(Park et al., 2017) [49]
	*Lactobacillus reuteri* HI120	HFD-fed C57BL/6J mice	12 weeks	↓ Serum TC levels in the HI120 group (↓ 47.82% vs. model) ↓ Serum TC levels in atorvastatin group (↓ 48.26% vs. model)	(Sun et al., 2020) [50]
	*Lactobacillus plantarum* Y15	HFD-fed C57BL/6J mice	4 weeks	↓ Serum TC, TG, and LDL-C levels (TC, TG, and LDL-C decreases were comparable to the pravastatin group)	(Liu et al., 2017) [40]
	*Lactobacillus paracasei* N1115	HFD-fed C57BL/6J mice	12 weeks	↓ Serum TC, TG, and LDL-C levels (TC: ↓ 46.79%; TG: ↓ 17.99%; LDL-C: ↓ 31.59% vs. model)	(Sun et al., 2023) [51]
	*Lactobacillus curvatus* HY7601, *Lactobacillus plantarum* KY1032	HFD-fed C57BL/6 mice	7 weeks	↓ Serum TC, TG, and LDL-C levels (TC: ↓ 20.1%; TG: ↓ 40.9%; LDL-C: ↓ 29.4% vs. model)	(Lee et al., 2024) [52]
	*Lactobacillus gasseri* RW2014	HFD-fed SD rats	6 weeks	↓ Serum TC, TG, and LDL-C levels (TC and TG decreases were comparable to the simvastatin group) ↑ Serum HDL-C levels	(Li et al., 2022) [53]
	*Enterococcus faecium* WEFA23	HFD-fed SD rats	35 days	↓ Serum TC, TG, and LDL-C levels (TC: ↓ 33.90%; TG: ↓ 35.17%; LDL-C: ↓ 30.02% vs. model) ↑ Serum HDL-C levels (HDL-C: ↑ 30.02% vs. model)	(Huang et al., 2018) [54]
	*Pediococcus acidilactici* FZU106	HFD-fed SD rats	8 weeks	↓ Serum TC, TG, and LDL-C levels (TC and LDL-C decreases were comparable to the simvastatin group) ↑ Serum HDL-C levels (HDL-C increase was comparable to the simvastatin group)	(Zhang et al., 2022) [55]
Clinical	*Lactobacillus paracasei* TISTR 2593	50 hyperlipidemic patients	90 days	↓ Serum LDL-C level (↓ 15.56% vs. placebo)	(Khongrum et al., 2023) [11]
	*Bifidobacterium longum* CCFM1077	62 hyperlipidemic patients	6 weeks	↓ Serum TC and LDL-C levels (TC: ↓ 5.7%; LDL-C ↓ 4.7% vs. placebo)	(Chu et al., 2023) [56]
	*Lactobacillus plantarum* ECGC 13110402	16 hypercholesterolemic adults, TC ≥ 6 mmol/L	6 weeks	↓ Serum TC and LDL-C levels (TC: ↓ 34.6%; LDL-C: ↓ 28.4% vs. before intervention)	(Keleszade et al., 2022) [41]
	*Lactobacillus plantarum* Q180	70 participants, TG < 200 mg/dL	12 weeks	↓ Serum LDL-C levels (LDL-C: ↓ 9.5% vs. placebo)	(Park et al., 2020) [57]
	*Lactoplantibacillus plantarum* CECT7527, *Lactoplantibacillus plantarum* CECT7528 and *Lactoplantibacillus plantarum* CECT7529	39 participants, TC ≥ 200 mg/dL	12 weeks	↓ Serum TC and LDL-C levels (TC: ↓ 11%; LDL-C: ↓ 13% vs. placebo)	(Guerrero-Bonmatty et al., 2020) [58]
	*Lactiplantibacillus casei* Zhang, *Bifidobacterium lactis* V9, *Bifidobacterium lactis* Probio-M8, *Lactiplantibacillus rhamnosus* Probio-M9, *Lactiplantibacillus plantarum* P-8	56 hyperlipidemic patients	3 months	↓ Serum LDL-C levels (LDL-C: ↓ 11.8% vs. placebo) ↑ Serum HDL-C levels (HDL-C: ↑ 9.5% vs. placebo)	(Wang et al., 2023) [12]

The arrows in the figure indicate the horizontal changes: ↑ represents an increase and ↓ represents a decrease.

**Table 2:** Qualitative determination of bile salt hydrolase activity.

Reference	Year	Method	Bile Salt
Bile salt hydrolase and lipase inhibitory activity in reconstituted skim milk fermented with lactic acid bacteria [37]	2021	Wells were drilled in MRS agar plates containing CaCl_2_ and 0.3% ox bile. Each well was inoculated with 25 µL of bacterial suspension and incubated under anaerobic conditions at 37 °C for 72 h.	0.3% ox bile
*Lactobacillus casei* YRL577 ameliorates markers of non-alcoholic fatty liver and alters the expression of genes within the intestinal bile acid pathway [66]	2021	MRS agar medium was supplemented with 0.3% taurodeoxycholate, 0.2% sodium thioglycolate, and 0.37 g/L of CaCl_2_. Sterile filter paper discs were evenly placed on the agar plate, and 10 µL of bacterial suspension was added to each disc; this was followed by incubation at 37 °C for 48 h.	0.3% taurodeoxycholate (TDCA)
Cholesterol-lowering potentials of lactic acid bacteria based on bile-salt hydrolase activity and effect of potent strains on cholesterol metabolism in vitro and in vivo [67]	2014	MRS agar plates containing 0.5% (*w*/*v*) sodium tauro (deoxy) cholate and 0.37 g/L of CaCl_2_ were prepared. Sterile filter discs (6 mm) were soaked in overnight bacterial cultures and placed on the agar plates; this was followed by anaerobic incubation at 37 °C for 72 h.	0.5% tauro (deoxy) cholate

## Data Availability

No new data were created or analyzed in this study. Data sharing is not applicable to this article.

## Supplementary Materials

The following supporting information can be downloaded at https://www.mdpi.com/article/10.3390/foods14091583/s1, Table S1: Risk of bias summary judgements about each risk of bias item for each included clinical study; Table S2: Risk of bias summary judgements about each risk of bias item for each included animal study.
