# Supplementary material for: Research Progress on the Mechanism of Action and Screening Methods of Probiotics for Lowering Blood Lipid Levels
Source: Foods. 2025 Apr 30;14(9):1583. doi: 10.3390/foods14091583 (PMC12071596; doi:10.3390/foods14091583)
Supplement: Supplementary file 1 [file foods-14-01583-s001.zip › foods-3525232-supplementary.pdf]

Supplementary Table S1. Risk of bias summary Judgements about each risk of bias item for each included clinical study.

Study	Year	Randomization process	Deviations from intended interventions	Missing outcome data	Measurement of the outcome	Selective reporting
Khongrum et al.	2023	unclear	low risk	low risk	unclear	low risk
Chu et al.	2023	low risk	low risk	unclear	low risk	low risk
Keleszade et al.	2022	unclear	low risk	low risk	low risk	unclear
Park et al.	2020	unclear	low risk	low risk	low risk	unclear
Guerrero-Bonmatty et al.	2020	unclear	low risk	low risk	low risk	unclear
Wang et al.	2023	unclear	low risk	unclear	unclear	low risk

Supplementary Table S2. Risk of bias summary Judgements about each risk of bias item for each included animal study.

Study	Year	Selection bias			Performance bias		Detection bias		Attrition bias	Reporting bias	Other biases
		Sequence generation	Baseline characteristics	Allocation concealment	Random housing	Blinding	Random outcome assessment	Blinding	Incomplete outcome data	Selective outcome reporting	Other sources of reporting bias
Park et al.	2017	unclear	low risk	unclear	unclear	unclear	unclear	unclear	low risk	unclear	low risk
Sun et al.	2020	unclear	unclear	unclear	unclear	unclear	unclear	unclear	low risk	unclear	low risk
Liu et al.	2017	unclear	unclear	unclear	unclear	unclear	unclear	unclear	low risk	unclear	low risk
Sun et al.	2023	unclear	unclear	unclear	unclear	unclear	unclear	unclear	low risk	unclear	low risk
Lee et al.	2024	unclear	unclear	unclear	low risk	unclear	unclear	unclear	low risk	unclear	high risk
Li et al.	2022	unclear	unclear	unclear	low risk	unclear	unclear	unclear	unclear	unclear	low risk
Huang et al.	2018	unclear	unclear	unclear	low risk	unclear	unclear	unclear	unclear	unclear	high risk
Zhang et al.	2022	unclear	low risk	unclear	unclear	unclear	unclear	unclear	low risk	unclear	unclear
